# Characteristics of Occupational Burnout among Nurses of Various Specialties and in the Time of the COVID-19 Pandemic—Review

**DOI:** 10.3390/ijerph192113775

**Published:** 2022-10-23

**Authors:** Robert Ślusarz, Klaudia Cwiekala-Lewis, Mariusz Wysokiński, Karolina Filipska-Blejder, Wiesław Fidecki, Monika Biercewicz

**Affiliations:** 1 Neurological and Neurosurgical Nursing Department, Faculty of Health Science, Collegium Medicum in Bydgoszcz, Nicolaus Copernicus University in Toruń, 85-821 Bydgoszcz, Poland; 2 School of Nursing & Health Professions, York College of Pennsylvania, York, PA 17403-3651, USA; 3 Department of Basic Nursing, Chair of Development in Nursing, Faculty of Health Sciences, Medical University, 20-081 Lublin, Poland; 4 Clinic of Geriatrics, Faculty of Health Science, Collegium Medicum in Bydgoszcz, Nicolaus Copernicus University in Toruń, 85-094 Bydgoszcz, Poland

**Keywords:** occupational burnout, nurses, risk factors, COVID-19 pandemic

## Abstract

Occupational burnout is particularly common among nurses due to their work being associated with stress, showing understanding, compassion, and commitment, along with the simultaneous need to maintain the necessary emotional distance. The aim of this review was to assess the occurrence and characterization of burnout among nurses working within neurology, geriatric care, intensive care units and with patients infected with the novel COVID-19 virus. PRISMA guidelines were used to conduct the review. The search for literature was limited to articles meeting the inclusion criteria and published from 2017 to 2022 in PubMed, Scopus, Science Direct, Google Scholar, and Wiley. A total of 768 articles from this category have been found. Ultimately, after in-depth analysis, 20 articles were included in the study. The group of respondents ranged from 49 to 3100 participants. According to the data, the percentages of nurses suffering from burnout in the presented research ranged from 14.3% to 84.7%, with the highest value of burnout among nurses who worked in the ICU during the COVID-19 pandemic. There are certain factors among nurses that significantly affect the occurrence of burnout. These include, among others, working time, age, exposure to infection and contact with infected patients, lack of training on COVID-19 prevention, providing care to an increased number of COVID-19 patients per shift, lack of personal protective equipment, lack of support of administration, lack of pay satisfaction, intrinsic motivation and turnover intention.

## 1. Introduction

The problem of burnout results from the importance that modern society assigns to work and its derivatives. It is often a consequence of excessive professional involvement along with the lack of positive feedback from the environment. The very term “burnout” accurately reflects the essence of exhaustion experienced by an individual as a result of highly stressful working conditions. This phenomenon occurs in people working in people-oriented professions focused on helping others. Burnout is mainly observed in helping relationships, which is why it occurs mainly in health care workers. It particularly often concerns nurses, because their professional work is associated with stress, showing understanding, compassion, and commitment, with the simultaneous need to maintain the necessary emotional distance [1,2].

Work overload and conflict, as well as scarcity of resources such as social support, professional skills and autonomy, and participation in decision making play a fundamental role in the development and course of burnout [3]. The consequences can be very serious and take many forms, from a decline in the sense of belonging to a given employer or organization, to dissatisfaction, staff turnover and absence at work. Burnout is revealed by a reduction in the quality of service provided to clients. From an individual perspective, costs translate into health problems, and often into substance abuse and/or family conflicts [4,5].

The World Health Organization (WHO), in the 11th revision of the International Classification of Diseases (ICD-11), defines occupational burnout (OB) as “a syndrome resulting from chronic workplace stress that has not been successfully managed” [6,7,8]. According to Christina Maslach, burnout is the body’s response to prolonged stress. We define burnout as a psychological syndrome of emotional exhaustion, depersonalization and a reduced sense of personal achievement that can occur in people who work with other people in a certain way. Emotional exhaustion refers to a person’s well-being, indicating that they are mentally overloaded, and their emotional levels have been significantly depleted. Depersonalization refers to a negative or overly indifferent reaction to other people who are usually recipients of the person’s services or the subject of his/her care. A reduced sense of personal achievement refers to a decline in self-competence and work success [9,10,11].

Nursing is one of the professions in which stress is aninherent property and results from the very nature of the profession. The work of nurses carries special psychological burdens, the source of which is another person, often in a difficult situation. The nurse works under strong and prolonged emotional tension [12,13]. In extreme situations, the lack of coping with stress, the characteristics and difficult of work in a given ward and the lack of team support lead to the formation of a burnout syndrome, which not only significantly reduces the quality of work, but also prevents nurses from further professional development [14]. Thus, in our manuscript, we conducted a review of studies on the prevalence and characteristics of burnout among nurses working in a neurology, geriatric, intensive care units and with patients infected with the COVID-19 virus.

## 2. Materials and Methods

### 2.1. Search Strategy

The full project procedure was carried out in accordance with the guidelines for the preferred reporting items for systematic reviews and meta-analyses (PRISMA) [15]. The appointed team of experts with experience in conducting this type of research and having methodological knowledge started the analysis and review of the literature using the following databases: PubMed, Scopus, Science Direct, Google Scholar, and Wiley. Keywords used in the project are job burnout, quality of work-life, nurses, influencing factors, department of neurology, intensive care unit, department of geriatric, COVID-19. The review of bibliographic databases lasted from March 2017 to September 2022.

### 2.2. Eligibility Criteria

The paper includes articles that met the following inclusion criteria: (1) presentation of original results, (2) published full reports and research results, (3) study group—nurses, (4) reporting short- and/or long-term outcome, (5) quantitative studies, (6) publication date from 2017 to 2022, (7) research using psychometric tools with high measurement reliability, (8) research conducted among nurses employed in one of the following departments: neurology, geriatric, intensive care unit, patients with COVID-19, (9) publications were in English. The exclusion criteria were: (1) case studies, short reports, (2) article written in different language than English, (3) small sample size < 49.

### 2.3. Study Selection

Two independent researchers conducted a literature review based on titles and abstracts. Articles that met the inclusion criteria were initially qualified for the analysis. Then, subsequent investigators reviewed and re-selected the chosen studies and removed any existing duplicates. Articles found to be irrelevant to the purpose of this analysis were excluded. The next stage was the full-text analysis of selected articles by other independent researchers. Data was extracted, followed by data synthesis and analysis. A total of 768 articles from this category have been found. Ultimately, after in-depth analysis, 20 articles were included in the study.

### 2.4. Data Extraction

The following data were collected and presented from the qualified studies: (1) the name and surname of the authors along with the publication date, (2) characteristics of the population, (3) age of the respondents, (4) place and/or form of the research, (5) tools used in the research, (6) the occurrence of occupational burnout with a short description, (7) a detailed description of the risk factors.

## 3. Results

### 3.1. Overview of Studies

**Figure 1:**
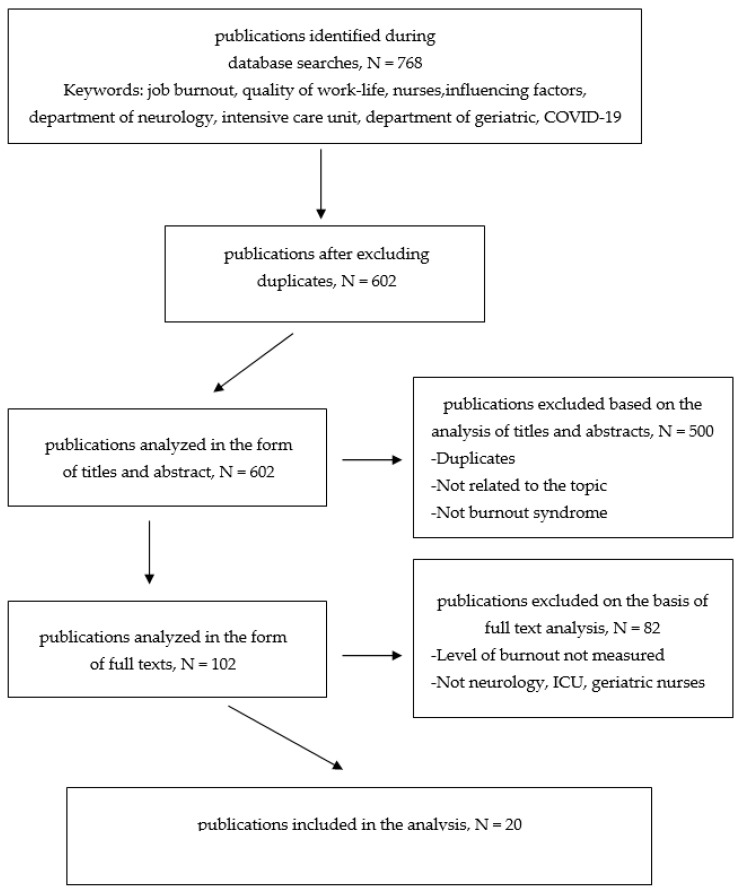
Pathways diagram of the literature search strategy.

As shown in Figure 1, 768 records were found when searching 5 databases. After excluding duplicates, 602 articles remained. Then, an analysis was performed on the basis of titles and abstracts, eliminating 500 articles. 102 articles were analyzed in full text. 20 studies were found to match the criteria presented and included in the review [16].

The manuscripts published in 2017–2022 were analyzed. The researchers aimed to present the most realistic and real research results, therefore only articles published within the last 6 years were taken into account. The group of examined people ranged from 49 [17] to 3100 [18] participants. Some studies qualified for the analysis published the results on medical personnel broken down by individual professions. Detailed characteristics of the studies is presented in Table 1.

### 3.2. Measurements and Tools

Various tools were used to assess the occurrence and characteristics of occupational burnout. The most frequently used scales were the Maslach Burnout Inventory-Human Services Survey (MBI-HSS) [18,19,20,21,22,23] and the Maslach Burnout Inventory (MBI) [24,25,26,27,28,29]. The most common form of research was an Internet survey [2,17,18,19,25,26,30,31,32,33].

### 3.3. Study Outcome

In the presented research, the results, and the way they are expressed differ depending on the tool used- as well as the design and vision of the authors. The percentages of occupational burnout among nurses within the presented studies ranged from 14.3% [20] to 84.7%, with the highest value of burnout reported among nurses who worked in the ICU during the COVID-19 pandemic [32]. In turn, in the studies by Kim and Yeom [34], the mean burnout score was 3.18 out of 5 (range 1.65–5). Wang et al. [23] noted occupational burnout at the level of 3.58 ± 2.55 (mean ± SD). On the other hand, Sarabia-Cobo et al. [2] and Iecovich and Avivi [28] determined the following mean burnout scores using the MBI scale: 26.71 ± 7.23 and 56.99 ± 18.07, respectively. From the conducted analysis, it can be concluded that the highest results of occupational burnout were recorded in nurses working during the COVID-19 pandemic.

Mean values of emotional exhaustion ranged from 1.32 ± 1.12 (mean ± SD) [23] to 25.4 ± 11.2 [18]. In studies by Purvis et al. [17] high emotional exhaustion was reported in 45% of the surveyed nurses working in the neurological ward. In turn, Alvares et al. [20] showed such high values of emotional exhaustion in 25% of nurses working in the Intensive Care Unit. Nurses working during the COVID-19 pandemic were also definitely in the highest percentage, who struggled with the problems of emotional exhaustion—37.2% [22], 36.9% [25]. Such high values were also noted among nurses working in the geriatric ward—37.2% [2] and 29.2% [29]. On the other hand, mean values of depersonalization ranged from 0.76 ± 0.94 [23] to 9.0 ± 6.3 [18]. In studies by Purvis et al. [17] and Fargen et al. [30] conducted among neurological nurses, the median of depersonalization was: 3 (IQR 0–6)and 6 (IQR 2–11), respectively. High depersonalization was noted in the studies by Alvares et al. [21] among 25% of nurses working in ICU and Sarabia-Cobo et al. [2] among 21.8% of geriatric nurses. Mean values of personal accomplishment ranged from 29.8 ± 3.5 [24] do 38.74 ± 6.20 [26].

The review includes articles assessing burnout in various aspects, broken down into individual forms of burnout. For example, Howie-Esquivel et al. [33] in studies conducted among nurses working during the COVID-19 pandemic showed that the mean values of personal burnout were 51.7 ± 21.9, work-related burnout- 50.1 ± 27.8 and client (patient)-related burnout- 27.6 ± 21.3. Similar studies were carried out among nurses working in the neurological and neurosurgical departments by our Polish research team [13]. We found that nearly 32% of nurses suffered from work-related burnout, 44.2% from colleague-related burnout, 22.8% from patient-related burnout. Kim and Yeom [34] presented the results of physical, emotional and psychological burnout among ICU nurses and they were, respectively: 3.43 ± 0.63, 3.28 ± 0.49, 2.87 ± 0.56. We observed similarly presented results in the studies by Howie-Esquivel et al. [33] and they were, for personal burnout—51.7 ± 21.9, work-related burnout—50.1 ± 27.8, client (patient)-related burnout—27.6 ± 21.3. On the other hand, in the studies conducted by Bąk et al. [12] the incidence rate of fatigue symptoms was shown to be 69.3 ± 68.33.

### 3.4. Key Results

Many studies, apart from occupational burnout, also assessed the occurrence of depressive disorders, which statistically often coexisted together. Saposnik et al. [19] showed that depressive symptoms were observed in nearly 14% of nurses caring for patients with multiple sclerosis. In turn, Vasconcelos et al. [20] reported the occurrence of depressive disorders in 11% of nurses (intensive care nurses). Moreover, significantly high rates of depression appeared among nurses during the COVID-19 pandemic. In a study by Andlib et al. [22], Kim and Lee [25] and Guttormson et al. [32], depressive disorders were diagnosed among 45.5%, 31.5% and 44.6% of nurses, respectively.

Among neurological and neurosurgical nurses there are some common factors that significantly influence the occurrence of burnout. These predictors include, among others, the longer working time, the greater the risk of occupational burnout [12,13,17]. The age of the nurses also plays an important role. In our previous research [13] it was shown that nurses older than 54years experienced burnout most frequently. Also, Bąk et al. [12] observed the highest frequency of burnout in the group of people aged 46–55. On the other hand, among ICU nurses, a study by Kim and Yeom [34] showed that factors such as: had no religion (*p* = 0.006), not cared for dying patients previously (*p* = 0.037), no bereavement experience for family (*p* = 0.034) significantly influenced the occurrence of occupational burnout. See et al. [18] presented the protective effect against burnout in their project, including: having a religious background or belief- OR 0.79 (95%CI 0.65–0.97), *p* = 0.023; work life balance- OR 0.87 (95%CI 0.81–0.95), *p* = 0.001. In turn, in studies conducted among nurses during the COVID-19 pandemic, exposure to infection and contact with infected patients turned out to be the most common shared risk factor. In addition, significant predictors turned out to be: lack of training on COVID-19 prevention, provided care to an increased number of COVID-19 patients per shift [22] and lack of personal protective equipment [32]. To further add lack of support of administration is a risk factor of burnout, moral distress, and symptoms of posttraumatic stress disorder [32]. Howie-Esquivel et al., 2022 showed that 64.4% of nurses more stressed while seeing patients due to COVID-19 [33]. In a study by Wang et al. [23] among nurses working in geriatric wards, the following were considered significant predictors: pay satisfaction, intrinsic motivation, turnover intention. It has also been shown that factors, such as: professional experience among older people [26], higher levels of agism, working in a for-profit facility reported [28], consideration of people with dementia as aesthetically unacceptable, the notion that there is a stigma in community structures towards people with dementia [29] have a significant impact on the burnout of geriatric nurses.

## 4. Discussion

This study provides important information on burnout among nurses in selected hospital departments. The review prepared by us sheds light on the coexistence of depressive disorders, anxiety, fear and important factors influencing the occurrence of burnout. Overall, there are many common predictors of burnout among nurses of different specialties. As part of this review, we highlighted burnout as a common phenomenon, affecting a large population of nurses. Therefore, a detailed assessment of risk factors plays a major role in the analysis of this phenomenon and in the conducted prevention, educational programs, or interventions. An important role is also played by the standardization of methodological aspects of research on burnout.

It is important to introduce some permanent implications for nursing and health policy. Due to the high percentage of occupational burnout among nurses, it would be important to introduce cyclical monitoring of its intensity. This is especially true in the current era of the COVID-19 pandemic, which, according to the presented studies, has further aggravated the growing problem [22,25,31,32,33]. Understanding what factors contribute to the occurrence of burnout and what kind of support nurses expect from management and the employer should be a priority in future research. The provision of mental support for medical personnel also plays an important role, among others, through cooperation with a psychologist. It should be emphasized that the key role in the aspect of prevention and intervention will be played by cooperation and an attempt at mutual understanding between employers, management and medical staff [13,24,34].

The studies presented by us in Table 1 show the whole range of risk factors for burnout among nurses. Furthermore, it should be noted that some of the results are inconsistent with each other. In the studies by Bąk et al., 2018 [12] and Ślusarz et al., 2022 [13]—older nurses were more burned out and in the study, meanwhile, in the study by Kim and Yeom, 2018 [34] and Guttormson et al., 2022 [32], young age promoted burnout. The discrepancy in these data may result from the methodological differences of the research, the country in which the project was conducted, cultural differences, and the applied statistical tests. Moreover, researchers rarely focus solely on socio-demographic factors as the main variables influencing the occurrence of burnout in nursing population. It is assumed that these variables should be assessed in combination with others, because it is in correlation with variables such as, for example, work environment, that they will allow to show the correct picture of burnout. Increasingly, instead of on socio-demographic factors, scientists focus on psychological factors, such as the occurrence of depression [19,20,35]. Ezenwaji et al. [36] assessed the impact of socio-demographic factors on burnout. It was shown that only gender was found to be significantly associated with work-related stress. The sociodemographic factors were not significantly associated with burnout among the nurses. Gómez-Urquiza et al. [35] also highlight the problem of conflicting burnout data among different age groups. Many researchers indicate that it is mainly the work environment, work experience, workload, and the lack of proper cooperation with physicians and managers that significantly correlate with burnout [35,36,37]. The number of hours spent at work and lack of sleep also statistically significantly correlate with burnout. Furthermore, more errors in patient care were reported among tired nurses [37,38]. Nursing population experience high levels of burnout due to heavy workload, and because they are always in contact with patients, they are easily exposed to infection that can cause work-related health problems [39].

## 5. Conclusions

In conclusion, in nurses as a group there is an increased risk of burnout, which is often associated with depressive disorders, anxiety and stress. According to the data, even every third nurse may show symptoms of burnout. The COVID-19 pandemic contributed to an increase in burnout rates among medical personnel, mainly among nurses. There is a strong need for more research in this area to understand root causes and to develop programs and interventions.

## Figures and Tables

**Table 1:** Prevalence and characteristic of burnout in nurses.

Author, Year	Sample	Age	Place	Measure	Prevalence of Burnout	Predictors of Burnout
Department of Neurology or Neurosurgery						
Bąk et al., 2018 [12]	110 nurses	N/A	filled in the ward	ADQ, the Japanese Questionnaire	- the incidence rate of fatigue symptoms, mean ± SD—69.3 ± 68.33	➢ the age group 46–55 years—73.3 ± 74.2 ➢ having more than four children—86.6 ± 88.3 ➢ working in multiple shift—70.9 ± 70.0 ➢ working in more than one position—73.9 ± 72.9
Purvis et al., 2019 [17]	65 Neurosciences Critical Care Unit Staff—49 (75%) nurses, 75% female	mean ± SD—34 ± 10	the survey online	aMBI	- emotional exhaustion, median (IQR)—8 (6–11) -depersonalization, median (IQR)—3 (IQR 0–6) - personal accomplishment, median (IQR)—15 (IQR 13–16) -high emotional exhaustion—45% (n = 29) - high depersonalization—28% (n = 18) of participants	➢ longer time working in the NCCU (1–5 years vs. less than 1 year) was independently associated with higher emotional exhaustion scores (*p* = 0.012)
Fargen et al., 2020 [30]	129 nurses and 109 technologists	N/A	the survey online	MBI-HSS MP	- emotional exhaustion, median (IQR)—25 (15–35) - depersonalization, median (IQR)—6 (2–11), - personal accomplishment, median (IQR)—39 (35–43) -the burnout prevalence in nurse—50% - the burnout prevalence in technologists—53%	➢ feeling under-appreciated by hospital/department- coefficient 1.4, OR 4.10 (95%CI 2.07–8.31), *p* < 0.001 ➢ physicians more difficult to work with- coefficient 0.19, OR 1.21 (95%CI 1.00–1.48), *p* = 0.05 ➢ increasing effect of work on family life- coefficient 0.13, OR 1.14 (95%CI 1.01–1.30), *p* = 0.04
Saposnik et al., 2022 [19]	96 nurses, 91.7% female	mean ± SD—44.6 ± 9.8	the survey online	MBI-HSS	- severe burnout—16.7% - depressive symptoms—13.5%	➢ burnout was associated with higher risk of sick leave—OR 1.06 (95%CI 1.00, 1.13), *p* = 0.04)
Ślusarz et al., 2022 [13]	206 nurses, 95.1% female	≥25 years	self-filled in the ward	ADQ	- work-related burnout—32% - colleague-related burnout—44.2% - patient-related burnout—22.8%	➢ nurses older than 54 years—OR 4.80 (95%CI 1.16, 19.92), *p* = 0.03 ➢ 20–29 years of working experience—OR 2.78, (95%CI 0.97, 7.97), *p* = 0.05
**Intensive care unit**						
Kim and Yeom, 2018 [34]	318 nurses, 97.2% female	mean ± SD—29.79 ± 5.71	self-filled survey (collected in sealed envelopes)	Burnout Questionnaire	- the mean burnout score—3.18 out of 5 (range 1.65–5) - physical burnout—3.43 ± 0.63 - emotional burnout—3.28 ± 0.49 - psychological burnout—2.87 ± 0.56	➢ aged 21–29 years (*p* < 0.001), ➢ single (*p* = 0.002), ➢ less educated (*p* = 0.001), ➢ less experienced (*p* < 0.001) ➢ staff nurse position (*p* < 0.001), ➢ had no religion (*p* = 0.006), ➢ not cared for dying patients previously (*p* = 0.037) ➢ no be reavement experience for family (*p* = 0.034)
See et al., 2018 [18]	3100 nurses	N/A	the survey online	MBI-HSS	- overall high burnout—52.0% - emotional exhaustion subscale, score range 0–54, mean ±SD—25.4 ± 11.2 -depersonalization subscale, score range 0–30, mean ± SD—9.0 ± 6.3 - personal accomplishment subscale, score range 0–48, mean ± SD—32.5 ± 9.0	➢ bachelor’s degree vs. non-degree qualification—OR 1.33 (95%CI 1.10–1.61), *p* = 0.003 ➢ protective effect against burnout: having a religious background or belief—OR 0.79 (95%CI 0.65–0.97), *p* = 0.023; work-life balance—OR 0.87 (95%CI 0.81–0.95), *p* = 0.001
Vasconcelos et al., 2018 [20]	91 nurses, 89.0% female	mean ± SD—30.82 ± 6.42	data collection was done by the researcher, during the participants’ working hours	MBI-HSS, BDI	- burnout—14.3%, - symptoms of depression—11.0%	➢ the percentage of individuals with burnout was higher among those who presented depressive symptoms (40.0%)
Alvares et al., 2020 [21]	125 nurses, 90.6% female	mean ± SD—36.5 ± 8.2	interview	MBI-HSS	- burnout syndrome, according to the criteria of Grunfeld—44.7% - high emotional exhaustion—25% - high depersonalization—20% - high reduced personal accomplishment—7.8%	➢ 35 years of age reduced the chances of developing depersonalization (OR 0.02) and emotional exhaustion (OR 0.08) ➢ not exercising regularly increased the chance of developing a high level of emotional exhaustion (OR 11.01) ➢ nurses working in an infant ICU (OR 4.74) achieve the reduced sense of personal accomplishment was more likely than nurses working in a general ICU
Möller et al., 2021 [24]	- 180 professionals in the public hospital—138 nursing technicians and 42 nurses - 116 professionals in the private hospital—94 nursing technicians and 22 nurses; >60% female	- public hospital: nurses- 40.4 ± 8.0, nursing technicians- 44.26 ± 7.78 - private hospital: nurses- 34.1 ± 4.4, nursing technicians—38.01 ± 7.38	N/A	MBI	BURNOUT -public hospital: nurses—2.5%, nursing technicians—9.1% - private hospital: nurses—9.5%, nursing technicians—8.5% NURSES -in public hospital: professional fulfillment—31.1 ± 3.3, emotional exhaustion—20.8 ± 5.1, depersonalization—8.10 ± 2.64, in private hospital: professional fulfillment—29.8 ± 3.5, emotional exhaustion—23.8 ± 4.6, depersonalization—8.68 ± 2.59 NURSING TECHNICIANS - in public hospital: professional fulfillment—32.25 ± 3.47, emotional exhaustion—19.74 ± 6.10, depersonalization—8.00 ± 2.65, in private hospital: professional fulfillment—32.96 ± 3.43, emotional exhaustion—18.15 ± 4.88, depersonalization—7.79 ± 2.71	N/A
**Burnout during the COVID-19 pandemic**						
Teo et al., 2021 [31]	2744, 60% nurses (90% female)	34.75	the survey online	PSS-4-, GAD-7, Physician Work Life Scale	- job burnout—27% - stress—36% - anxiety—14%	➢ 5–9 years experience (OR 1.72, *p* < 0.05) ➢ long work hours (OR 4.12, *p* < 0.01) ➢ occasional COVID-19 contact (OR 1.37, *p* < 0.10) ➢ team work well together (OR0.55, *p* < 0.01) ➢ job dedication (OR 0.76, *p* < 0.01) ➢ feel appreciated at work (sometimes/always) (OR 0.34, *p* < 0.01) ➢ emotional support (OR 0.98, *p* < 0.05) ➢ self-efficacy (OR 0.96, *p* < 0.01)
Andlib et al., 2022 [22]	288 nurses, 76.7% female	mean ± SD—27.7 ± 4.4	self-filled survey at their convenient time and handed them over to their nurse managers	MBI-HSS, STOP-D	- overall burnout—48.6% - high levels of emotional exhaustion—37.2% - depersonalization—36.8% -low levels of personal accomplishment—46.9% - depression—45.5% - anxiety—48.6% - stress—47.2% - anger—43.7%	➢ employed in public hospitals (*p* < 0.001) ➢ lack of training on COVID-19 prevention (*p* = 0.005) ➢ worked for <48 h per week (*p* = 0.021) ➢ provided care to an increased number of COVID-19 patients per shift (*p* < 0.001)
Kim and Lee, 2022 [25]	111 nurses, 94.6% female	Age: 20 years—54.1%, 30 s—28.8%, 40 s—10.8%, 50 s—6.3%	the survey online	PHQ-9, GAD-7, IES-R, MBI	- burnout—44.1% - emotional exhaustion—36.9% -depersonalization—29.7% - personal accomplishment—36% - depression—31.5% - anxiety—8.1% - distress—23.4%	➢ performing COVID-19 tasks is associated with a higher incidence of moderate depression
Guttormson et al., 2022 [32]	488 nurses, 88.1% female	20–30 years—39.3%, 31–40 s—28.7%, 41–50 s—13.7%, 51–60 s—13.3%, >60 s—4.9%	the survey online	PROQOL-5, TSQ, PHQ-ADS	- moderate levels of burnout (Mdn. 30, range 10 to 44; 84.7% moderate levels) - moderate to severe anxiety—31.1% - moderate to severe depression—44.6% - risk for developing posttraumatic stress disorder—46.7%	➢ younger nurses (20–30 years old) were more likely to experience burnout than either nurses aged 41–50 (*p* = 0.031) or nurses aged 51–60 (*p* = 0.023) ➢ experience of five years or less had significantly higher reported burnout than more than 20 years of experience (*p* = 0.019) ➢ female more often than male experienced burnout (*p* = 0.005), posttraumatic stress disorder risk (*p* = 0.013), and anxiety (*p* = 0.014) ➢ lack of personal protective equipment had higher burnout (*p* < 0.001), moral distress (*p* < 0.001), anxiety (*p* = 0.004), depression (*p* = 0.010), and symptoms of posttraumatic stress disorder (*p* = 0.020). ➢ lack of support of administration is a risk factor of burnout, moral distress, and symptoms of posttraumatic stress disorder ➢ burnout and moral distress were both moderately to highly correlated with anxiety, depression, and posttraumatic stress disorder symptoms
Howie-Esquivel et al., 2022 [33]	101 nurses, 93% female	mean ± SD—50.2 ± 10.8	the survey online	CBI, WRQoL	- personal burnout, mean ± SD—51.7 ± 21.9 - work-related burnout, mean ± SD—50.1 ± 27.8 - client (patient)-related burnout, mean ± SD—27.6 ± 21.3	➢ 64.4% nurses more stressed while seeing patients due to COVID-19 ➢ general well being ➢ home-work interface ➢ job career satisfaction ➢ control at work ➢ working conditions ➢ stress at work ➢ overall quality of work life
**Geriatric ward**						
Potard and Landais, 2021 [26]	279 nurses and care assistants, 98.6% female	mean ± SD—36.00 ± 10.47	the survey online	MBI	- personal accomplishment, mean ± SD—38.74 ± 6.20, moderate burnout—21.86%, high burnout—31.9% - depersonalization, mean ± SD—7.34 ± 6.10, moderate burnout- 26.7%, high burnout—23.85% - emotional exhaustion, mean ± SD—22.61 ± 11.37, moderate burnout—27.55%, high burnout—34.41%	➢ age (*p* = 0.02) ➢ professional experience among older people (*p* = 0.05) ➢ professional experience in actual job (*p* = 0.01)
Wang et al., 2019 [23]	1212 nurses, 84% female	mean ± SD—49.16 ± 10.41	one-on-one interviews	MBI-HSS	- job burnout, mean ± SD—3.58 ± 2.55 - emotional exhaustion, mean ± SD—1.32 ± 1.12 - depersonalization, mean ± SD—0.76 ± 0.94 - reduced personal accomplishment, mean ± SD—1.50 ± 1.3	➢ pay satisfaction (correlation: −0.l07, *p* = 0.001) ➢ intrinsic motivation (correlation: −0.461, *p* = 0.001) ➢ turnover intention (correlation: 0.265, *p* = 0.001)
Sarabia-Cobo et al., 2021 [2]	281 nurses, 91% female	mean ± SD—36.8 ± 5.4	the survey online	AAQ-II, MBI, ProQOL	- burnout, mean ± SD—26.71 ± 7.23 - emotional exhaustion—high levels—37.2%, medium levels—47.6% - depersonalization—high levels—21.8% - performance at work—high levels—26.6%, medium levels—34.8%	➢ presenting compassion fatigue
Iecovich and Avivi, 2017 [28]	154 nurses, 74% female	mean ± SD—42.08 ± 11.00	self-administered questionnaire	MBI	burnout, mean ± SD—56.99 ± 18.07	➢ higher levels of agism ➢ lower levels of professional education ➢ fewer years working as a nurse ➢ working in a for-profit facility reported
Mantzorou et al., 2020 [29]	171 nurses and professional caregivers, 84.2% female	mean ± SD—37.5 ± 11.5	self-administered questionnaire	MBI	- emotional exhaustion—high level—29.2%, medium level—29.8% - depersonalization—high level—11.1%, medium level—25.1% - personal accomplishments—high level—35.1%, medium level—36.3%	➢ the desire to change their workplace ➢ consideration of people with dementia as aesthetically unacceptable ➢ feeling that their patients upset and annoy people around them ➢ the notion that there is a stigma in community structures towards people with dementia ➢ the feeling of shame and fear ➢ the perception that members of society see people with dementia as aesthetically unacceptable ➢ the willingness to help and the feeling that they really help with the activities of daily living ➢ the low degree of willingness to help and the low sense of assisting with daily life activities ➢ there porting of a small degree of cognitive dysfunction of their patient ➢ working in a nursing home 1–5 years versus a working experience under one year ➢ selection of working in a nursing home for other reasons and not for a livelihood

AAQ-II—Acceptance and Action Questionnaire II, ADQ—Author’s Questionnaire, aMBI—abbreviated Maslach Burnout Inventory, BDI—Beck Depression Inventory, CBI—Copenhagen Burnout Inventory, GAD-7—Generalized Anxiety Disorder-7,IES-R—Impact of Event Scale–Revised, MBI—Maslach Burnout Inventory, MBI-HSS—Maslach Burnout Inventory-Human Services Survey, MBI-HSS MP—Maslach Burnout Inventory-Human Services Survey for Medical Personnel, PHQ-9—Patient Health Questionnaire-9, PHQ-ADS—Patient Health Questionnaire Anxiety and Depression Scale, PSS-4—Perceived Stress Scale-4, ProQOL—Professional Quality of Life Scale (ProQOL), PROQOL-5—Professional Quality of Life Scale-5, STOP-D—Screening Tool of Psychological Distress, TSQ—Trauma Screening Questionnaire, WRQoL—Work-Related Quality of Life Scale.

## Data Availability

Not applicable.
